# Cognitive Foundations of Early Mathematics: Investigating the Unique Contributions of Numerical, Executive Function, and Spatial Skills

**DOI:** 10.3390/jintelligence11120221

**Published:** 2023-12-01

**Authors:** Hannah L. Whitehead, Zachary Hawes

**Affiliations:** Department of Applied Psychology & Human Development, Ontario Institute for Studies in Education, University of Toronto, Toronto, ON M5S 1V6, Canada; hannah.whitehead@mail.utoronto.ca

**Keywords:** spatial skills, numerical skills, executive function (EF) skills, mathematics, number line estimation, arithmetic, arithmetic strategies, spatial visualization

## Abstract

There is an emerging consensus that numerical, executive function (EF), and spatial skills are foundational to children’s mathematical learning and development. Moreover, each skill has been theorized to relate to mathematics for different reasons. Thus, it is possible that each cognitive construct is related to mathematics through distinct pathways. The present study tests this hypothesis. One-hundred and eighty 4- to 9-year-olds (*M*_age_ = 6.21) completed a battery of numerical, EF, spatial, and mathematics measures. Factor analyses revealed strong, but separable, relations between children’s numerical, EF, and spatial skills. Moreover, the three-factor model (i.e., modelling numerical, EF, and spatial skills as separate latent variables) fit the data better than a general intelligence (*g*-factor) model. While EF skills were the only unique predictor of number line performance, spatial skills were the only unique predictor of arithmetic (addition) performance. Additionally, spatial skills were related to the use of more advanced addition strategies (e.g., composition/decomposition and retrieval), which in turn were related to children’s overall arithmetic performance. That is, children’s strategy use fully mediated the relation between spatial skills and arithmetic performance. Taken together, these findings provide new insights into the cognitive foundations of early mathematics, with implications for assessment and instruction moving forward.

## 1. Introduction

Early mathematics knowledge is a strong predictor of future academic success, life opportunities, and well-being (Duncan et al. 2007; Reyna et al. 2009; Ritchie and Bates 2013). For example, mathematics skills at preschool have been shown to predict reading and mathematics performance in high school (Duncan et al. 2007; Watts et al. 2014). Numerical knowledge at seven is associated with one’s adult socioeconomic status (SES); a powerful correlate of many important life outcomes (Ritchie and Bates 2013). The importance of mathematics is further demonstrated by the fact that for thousands of years and throughout the world, the learning of mathematics has remained a central goal of education (Karp and Schubring 2014).

Against this background, there has been an increasing interest in better understanding the various factors (e.g., social, cultural, cognitive, etc.) that underlie and give rise to early mathematics skills and knowledge. A better understanding of these factors has the potential to provide key insights into how children learn mathematics and can be used to help inform assessment, instruction, and intervention. 

The present paper concerns itself with the cognitive foundations of early mathematics. We examine how numerical, executive function (EF), and spatial skills relate to one another and mathematics performance. Indeed, each one of these cognitive skills—numerical, EF, and spatial—has been shown to share strong concurrent and longitudinal relations with children’s mathematics performance (Atit et al. 2022; Schneider et al. 2017; Spiegel et al. 2021). Moreover, these relations exist for typical and atypical populations and across a wide age range (Cragg and Gilmore 2014; Hawes and Ansari 2020; Mix and Cheng 2012; Rousselle and Noël 2007; Toll et al. 2011). For this reason, these three cognitive skills represent ideal candidates in the effort to better understand the cognitive “building blocks” of early mathematics achievement. We review each one of these cognitive skills next, providing a brief review of the theoretical and empirically demonstrated links between each construct and mathematics performance. 

### 1.1. Numerical Skills and Mathematics

An understanding of numbers and their relations to one another is critical for mathematics. The theoretical link between early numerical skills and higher-level mathematics is straightforward: mathematics learning is cumulative in nature; therefore, basic numerical skills represent the foundation on which more advanced mathematics knowledge is built upon. For example, understanding cardinality (i.e., understanding the number of elements in a set) provides the foundation for engaging in basic addition strategies, such as counting on from the larger of two addends. 

Previous research has identified magnitude processing and ordinality as being two critical components of early numerical skills (Aunio and Niemivirta 2010; Goffin and Ansari 2016; Nosworthy et al. 2013). Magnitude processing, or one’s ability to discriminate between quantities, is typically measured by the speed and accuracy to which individuals can compare and select the largest of two numerical magnitudes (6 vs. 3 or ∷ vs. ∵). Such tasks have long been used to assess “number sense” and the precision of one’s mental representation of number (Dehaene 2011; Moyer and Landauer 1967; Siegler 2016). An extensive body of research suggests that children and adults who are faster and more accurate at comparing numerical symbols (6 vs. 3) and, to less of an extent, nonsymbolic numbers (∷ vs. ∵), tend to also perform better on higher-level mathematics tasks, such as arithmetic (see Schneider et al. 2017 for a meta-analysis). 

Ordinality, or one’s understanding of the order of number sequences, is another important indicator of an individual’s basic number skill. In general, children and adults who are faster and more accurate at identifying and producing ordered numerical sequences (e.g., 5-6-7) also tend to demonstrate higher-level mathematical skills (Lyons et al. 2014; Xu and LeFevre 2021). Moreover, ordinality skills have been found to predict mathematics achievement independent of magnitude processing skills (Goffin and Ansari 2016). 

In short, children’s magnitude and ordinality skills represent foundational numerical skills and are considered precursors to more advanced mathematical reasoning, including arithmetic. It is for this reason that the present study included these measures—magnitude comparison and ordinality—as key indicators of children’s basic numerical skill. 

### 1.2. Executive Function and Mathematics

In general, stronger EF skills have been associated with stronger mathematics performance (Cragg and Gilmore 2014; Emslander and Scherer 2022; Spiegel et al. 2021). The reasons for this link are varied and appear to depend on the EF and mathematics skills under question. One’s definition of EF also matters. Here, we defined EF using Miyake et al.’s (2000) tripartite model, that is, as a cognitive construct that includes three highly related yet separable cognitive capacities, including working memory, inhibitory control, and flexible attention (see also Friedman and Miyake 2017). Each one of these components has been linked to mathematics performance for different reasons (Cragg et al. 2017; Cragg and Gilmore 2014). That said, many studies find that EF is better represented by a one-factor model in childhood (Brydges et al. 2012; Duncan et al. 2016; Wiebe et al. 2011; Willoughby et al. 2012). It is this framework that guides the current study, as we combine EF skills into one overall latent EF factor. For example, when solving a mental arithmetic problem with multiple components (e.g., 15 + 8 − 2), a child would be required to hold the different components of the problem in mind and switch between different strategies or mental operations, requiring multiple aspects of EF within the same problem.

Taken together, there is both strong theoretical and empirical evidence to suggest that EF skills play a strong supporting role in mathematical reasoning. In the present study, we measured EF as a single construct, measured using a visual–spatial working memory task (forward and backwards path span) and a more holistic measure (head–toes–knees–shoulders task) which requires all three components of EF (McClelland and Cameron 2012; Miyake et al. 2000). 

### 1.3. Spatial Skills and Mathematics

A large body of evidence indicates consistent and robust associations between spatial and mathematical thinking (e.g., see Atit et al. 2022; Mix and Cheng 2012). A meta-analysis by Hawes et al. (2022) further suggests a causal relation between the two. It is important to acknowledge, however, that both spatial thinking and mathematics are multidimensional constructs. Thus, the relations that exist between the two may depend on the spatial skills and mathematics in question. Spatial visualization skills, defined as the ability to generate, retrieve, maintain, and manipulate visual–spatial information (Lohman 1996), appear to play a particularly important role in mathematics learning and performance. Indeed, spatial visualization skills have been linked to success across a wide range of mathematical domains, spanning basic numerical processing and arithmetic all the way up to highly advanced mathematics (Hawes et al. 2019; Casey et al. 2015; Wei et al. 2012). 

Why are spatial and mathematical thinking related? Several accounts have been put forward (e.g., see Hawes and Ansari 2020; Mix 2019). According to the *spatial modeling account*, spatial thinking and mathematics are linked through the affordances of spatial visualization; that is, spatial visualization may serve as a “mental blackboard” on which various mathematical concepts, relations, and operations can be modeled and visualized (Lourenco et al. 2018). For example, individuals may draw on their visualization skills to organize and model the various elements of a word problem. According to the *spatial representation of numbers account*, numbers are represented spatially, from smallest to largest, left-to-right (at least in cultures that read/write left-to-right), along a “mental number line” (Dehaene et al. 1993; Patro et al. 2016). A common index of one’s mental number line involves presenting individuals with an empty number line (i.e., a horizontal line flanked with 0 at the far-left end and another number, such as 100, flanked at the other end) and having them estimate the exact location of a given number (e.g., Where does 67 belong?). Lastly, according to the *working memory account*, the relation between spatial skills and mathematics may be due to other variables, such as working memory, EFs, or general intelligence, which share and explain the majority of variance between spatial and mathematical thinking. For example, it is possible that EF skills, including visual–spatial working memory, fully account for the relations previously observed between spatial visualization and mathematics performance. A central aim of the present study is to provide new insights into the three mechanisms just described.

To summarize, there are both theoretical and empirical reasons why spatial visualization may play an important role in mathematics learning and performance. For this reason, spatial visualization was targeted as the spatial skill of interest in the current investigation. 

### 1.4. Unique Contributions of Numerical, Executive Function, and Spatial Skills 

As just revealed, current evidence indicates that numerical, EF, and spatial skills each play an important role in mathematics learning and performance. Moreover, each skill appears to be related to mathematics for different reasons. Thus, it is possible that each cognitive construct is related to mathematics through distinct pathways. At the same time, there is also evidence to suggest that that these three variables may be highly related to one another, and in some cases, indistinguishable from one another, or perhaps better accounted for by a single latent intelligence factor (i.e., *g*). For example, Miyake et al. (2001) found that latent factors representative of spatial visualization and EF skill were very highly related to one another. There is very little work investigating the unique contributions of all three skills within the same model. As such, little is known about whether and how these foundational skills are related to one another at the latent level and whether each is *uniquely* related to mathematics achievement.

One exception—and one the present study aims to extend—is a study by Hawes et al. 2019. They found that while numerical, EF, and spatial skills all represented separate constructs, only numerical and spatial skills were uniquely predictive of mathematics achievement, with especially strong relations between spatial visualization skills and mathematics. In that study, mathematics was assessed using two broad-scale measures of numeration and geometry. However, given the multifaceted nature of mathematics, these two measures are certainly not representative of all mathematics achievement. As noted above, the relations between these variables (numerical, EF, and spatial skills) may depend on the mathematics at hand. For example, spatial visualization skills have been found to share stronger associations with mathematics problems that are novel and unfamiliar to the learner (Mix et al. 2016). Indeed, this was offered as one of the explanations for why spatial visualization was found to be the most robust correlate of mathematics achievement in the Hawes et al. (2019) study; both mathematics measures featured applied, novel problems. 

Among the unique contributions of the current study is the focus on more targeted measures of mathematics achievement: number line estimation and arithmetic. These outcome measures differ from the mathematics outcomes measures used in Hawes et al. (2019) in two important ways. First, they target more familiar mathematical content and procedures. Second, they contain less overtly spatial mathematics problems: both the numeration and geometry measures used in the 2019 study involved the presentation of items through visual–spatial representations (e.g., reasoning about graphs, shapes, arrays, etc.), with many items requiring explicit spatial reasoning (e.g., identifying a figure given the front, side, and top views). Together, these two factors may have led to an overestimation of the importance of spatial skills for mathematics learning and performance. 

### 1.5. Current Study

The present study examines the relations between numerical, EF, spatial skills and mathematics. A central aim is to test the extent to which the findings observed in the Hawes et al. (2019) study extend to different measures of mathematics used in the current study. Whereas Hawes et al. (2019) found that spatial and, to a lesser extent, numerical skills were unique predictors of mathematics, it remains possible that these results were due to the types of mathematics assessments used. Indeed, there is reason to predict that each foundational skill—numerical, EF, and spatial—provides unique and important pathways to mathematics success. The present study aims to tests this possibility, providing further insight into the cognitive building blocks of early mathematics.

## 2. Materials and Methods

### 2.1. Participants

One-hundred and eighty 4- to 9-year-olds (Kindergarten—Grade 3) participated in the study (mean age = 6.21, SD = 1.38; girls = 96, boys = 84). A portion of these participants (65%) also participated in Hawes et al. (2019). The sample was drawn from three urban elementary schools in Southwestern Ontario, Canada. All three schools are located in low-income neighborhoods (based on Canadian census data) and were considered low-performing schools in mathematics at the time of this study (according to standardized provincial test score data). Written consent was provided by a parent/guardian for all children, and research was carried out in agreement with the ethics boards of the University of Toronto, University of Western Ontario, and the local public school board. 

### 2.2. Measures and Procedure

A shown in Table 1, participants completed a total of 11 measures. Measures were completed in pseudo-random order. That is, certain measures were presented in ordered blocks: symbolic number comparison, nonsymbolic number comparison, and ordering; path span forward and path span reverse. Measures were completed in two approximately 30 min sessions, between 1 and 5 days apart. All measures were administered one-on-one with a trained experimenter in a quiet location within the child’s school (e.g., empty classrooms or private testing rooms).

#### 2.2.1. Numerical Assessments

For all numerical assessments, children were given 1 min to complete as many items as possible. Scores were derived by subtracting the total number of errors from the total number of correct responses. For additional details regarding the development of these measures and their validity in assessing mathematics, see Nosworthy et al. (2013). 

*Symbolic comparison:* Children crossed off the larger of two adjacent symbolic numbers as quickly and accurately as possible (e.g., 6 vs. 3). The task consisted of 72 items. 

*Nonsymbolic comparison:* Children crossed out the larger of two dot adjacent arrays as quickly and accurately as possible (e.g., six dots vs. three dots). The task consisted of 72 items. 

*Ordering task:* Children were presented with a numerical sequence (e.g., 3-4-5) and indicated whether or not the sequence was in order (i.e., ascending, from smaller to larger numbers). Children put a line through a checkmark to indicate sequences believed to be in the correct order and a line through an ‘X’ when the order was believed to be in the wrong order. The task consisted of 48 items (for more details on this task, see Hutchison et al. 2022). 

#### 2.2.2. Spatial Assessments

*2-D Mental Rotation Task:* This measure was adapted from the Children’s Mental Transformation Task (Levine et al. 1999), a widely used measure of children’s mental rotation skills (e.g., see Ehrlich et al. 2006; Hawes et al. 2015). The measure consisted of 16 items. For each item, children were presented with two halves of a 2D shape, which had been separated and rotated 60° from one another on either the same plane (direct rotation items) or diagonal plane (diagonal rotation items). Children were also shown four other shapes and asked to indicate which of the shapes can be made by putting the two halves together. Children were awarded a point for each correct response and a total score out of 16. 

*Visual–Spatial Reasoning Task:* This measure was adopted from Hawes et al. (2017) and provides a comprehensive measure of children’s spatial visualization skills. The measure consisted of 20 items divided into four types of spatial visualization problems: missing puzzle pieces (two variations), mental paper folding, and composition/decomposition of 2D shapes. In each problem, children were presented with the problem and asked to identify the correct answer among four options. Children were awarded a point for each correct response and a total score out of 20. 

*Raven’s Matrices:* This is a widely used measure of children’s visual–spatial analogical reasoning and general intelligence (Raven 2008). Previous research has found that performance on the task is closely linked to the latent construct of spatial visualization (e.g., see Hawes et al. 2019; Lynn et al. 2004). For each item, children were presented with a partially completed visual–spatial pattern and asked to identify from six options the puzzle piece that completes the pattern. The measure consisted of 36 items; children were awarded one point for each correct response. 

#### 2.2.3. Executive Function

*Head–Toes–Knees–Shoulders Task:* This measure was adapted from Ponitz et al. (2009). The task requires children to engage in flexible attention, working memory, and inhibitory control (McClelland and Cameron 2012), three core components of executive functioning (e.g., see Miyake et al. 2000). Children listen to a command and are asked to perform the paired “opposite” action; head and toes represent one pair, and knees and shoulders represent the other pair. For example, if the command is “touch your head”, the correct response would be to touch their toes, and if the command is “touch your shoulders”, the correct response would be to touch their knees. The task consisted of two sections, and each section consisted of 10 items. In the first section, children were only given one pairing (head–toes or shoulders–knees), but in the second section, they received commands for both pairings. For each item, children were given a score of 0, 1, or 2; 0 points if they performed the incorrect action, 1 point if they motioned towards the incorrect action but self-corrected, and 2 points if they performed the correct action. Children were given a total score out of 40. 

*Forward Path Span:* This task was administered on an iPad and used to measure children’s working memory. Children were presented with sets of nine randomly arranged green circles and instructed to watch as the circles lit up one at a time (each for 0.6 s, with 0.5 s between presentation). Children were then asked to tap the circles in the same order that they were presented. Following a practice trial, children began with two trials at a sequence length of two. If the child successfully recalled one or two sequences of a given length, they progressed to the next sequence length (e.g., if the child correctly repeated a sequence length of two on one or two trials, they were presented with two trials at a sequence length of three). The task was discontinued when the child failed to get two sequences of any given length. Scores were based on the total number of correct sequences recalled. For more information and to access the measure, see https://hume.ca/ix/pathspan.html (accessed on 15 September 2016).

*Reverse Path Span*: The task is the same as the Forward Path Span but required children to recall the sequences in reverse order in which they were presented. Scores were based on the total number of correctly reversed sequences recalled. For more information and to access the measure, see https://hume.ca/ix/pathspan.html (accessed on 15 September 2016).

#### 2.2.4. Mathematics Achievement

*Number Line Estimation:* This task was administered on an iPad (to access the application, see: https://hume.ca/ix/estimationline.html, accessed on 15 September 2016) and used to measure children’s numerical estimation performance, a strong and reliable predictor of broader mathematical competence (Schneider et al. 2018). Children were presented with a horizontal line marked with ‘0’ at the far-left end of the line and either ‘10’ (for children in Kindergarten) or ‘100’ (for children in Grades 1–3) at the far-right end of the line. Children are then asked to indicate where a given number belongs on the number line (e.g., where does the 7 go?). Children were first presented with a practice trial. For kindergarten children, the practice trial involved the placement of ‘5’, and for children in Grades 1–3, the practice trial involved the placement of ‘50’. The test trials for kindergarten children included numbers 1–9 (with the exception of 5). For children in Grades 1–3, test trials included the following target numbers adopted from Laski and Siegler (2007): 2, 3, 5, 8, 12, 17, 21, 26, 34, 39, 42, 46, 54, 58, 61, 67, 73, 78, 82, 89, 92, and 97. All trials were randomly presented to children. The accuracy of each trial was recorded by the computer. We then used this information and the following formula to calculate each child’s percent absolute error (PAE) across all trials. A lower PAE indicates less error.

$$PAE=\frac{Estimate-Estimated\;Quantity}{Scale\;of\;Estimates}100$$

*Mental Arithmetic and Strategy Use:* Children were aurally presented with twelve single-digit addition problems of increasing difficulty. The first four problems involved solutions with sums of five or less, the next four problems involved solutions between 6 and 10, and the last four problems involved solutions between 11 and 15. Items were counterbalanced so that half started with the smaller addend first and the other half started with the larger addend first. Children were awarded 1 point for each correct response and given a total score out of 12 (for further details, see Hawes et al. 2021).

Children’s strategy use was recorded for each question and based on observation and self-report (“Can you tell me how you got that answer?”; see Siegler 1987). In cases where children’s self-report contradicted their observed strategies, observed overt behaviors were given preference. Consistent with previous literature (e.g., Siegler 1987), the following behaviors/strategies were used and reported on by children: guessing, counting up (also known as count-all), counting on from the smaller addend, counting on from the larger addend, composition/decomposition, and automatic retrieval. Counting up refers to counting both addends separately prior to adding them together. Counting on refers to beginning with one addend and counting on from there. Composition/decomposition refers to deconstructing a problem into simpler parts (e.g., to solve 5 + 6, one might first add 5 + 5 and then add 1). Automatic retrieval refers to quick access to solutions committed to memory. Composition/decomposition strategies and automatic retrieval are considered more advanced than the other strategies and associated with superior arithmetic performance (Casey et al. 2017). 

Children’s strategy scores were calculated in two different ways depending on the analysis used. In the mediation model, strategy scores were treated as a continuous variable. That is, for each item children were assigned a score of 0–6, based on the sophistication of each strategy (0 = I don’t know/no guess; 1 = Guess; 2 = Count up strategy, 3 = Count on from smaller addend; 4 = Count on from larger addend; 5 = Composition/decomposition strategy; 6 = Automatic retrieval). Using this approach, each child was awarded a total arithmetic strategy score out of 72 with higher scores indicating more frequent use of more sophisticated strategies. We also calculated the percentage of times a child used a given strategy. For example, a child who relied on automatic retrieval for 50% of the items would have an automatic retrieval score of 50. This scoring method was used for our analyses examining the relations between each factor and the likelihood of using each strategy. 

### 2.3. Analytical Approach

Structural equation model analyses were carried out using the lavaan package (Rosseel 2012), and correlations were carried out using the corrplot package (Wei and Simko 2021), both of which are in R Studio (RStudio Team 2022). The recommended two-step approach to structural equation modeling (SEM) was followed (Kline 2015). In the first step, confirmatory factor analysis was used to construct a measurement model; the measurement model included latent constructs for numerical, executive function, and spatial skills. Following the measurement model, we constructed a structural model to investigate how these latent constructs were associated with each other and to each of the mathematics outcome measures. Note that it was our original intention to also treat number line estimation and arithmetic as a latent ‘mathematics’ variable. However, the fit statistics for a latent mathematics variable were poor, suggesting the need to treat number line estimation and arithmetic as separately manifested variables of mathematics performance. 

Three goodness-of-fit statistics were used to determine model fit: (1) Root Mean Root Mean Square Error of Approximation (RMSEA), (2) Comparative Fit Index (CFI), and (3) Standardized Root Mean Residual (SRMR). Model fit was deemed ‘good’ or acceptable if it met the following criteria: RMSEA values of <0.10, CFI values > 0.95, and SRMR values < 0.08 (Kline 2015).

Missing data were treated using listwise deletion, the default approach in the lavaan package. This resulted in a total of 172 participants for the model where number line estimation was the outcome variable and a total of 166 participants for the model where arithmetic performance and strategy use were the outcome measures (see Table 2 for details on missing data across measures). 

## 3. Results

### 3.1. Preliminary Analyses

**Figure 1:**
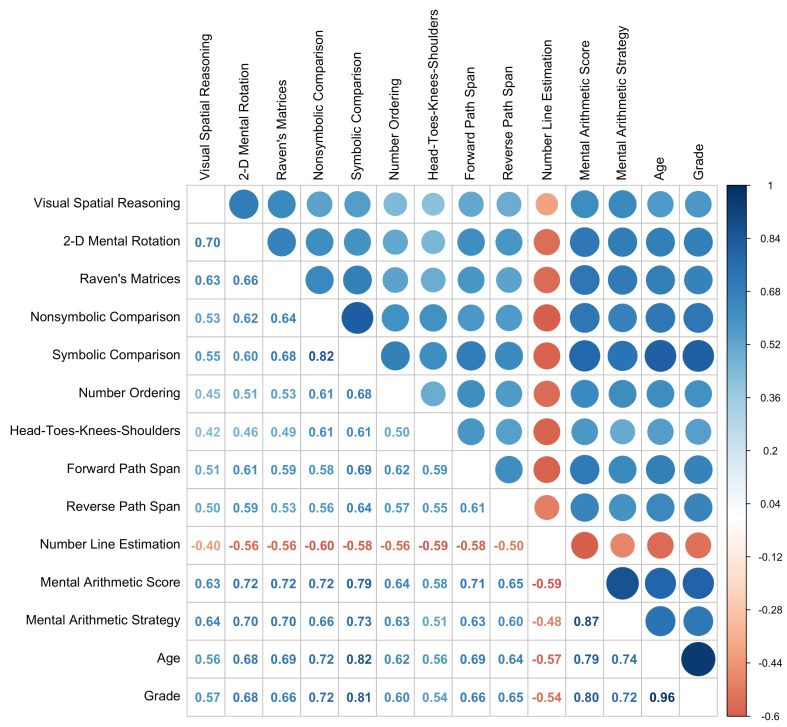
Correlations between spatial, numerical, EF, mathematics, age, and grade variables.

Descriptive statistics for each measure are shown in Table 2. Figure 1 shows the zero-order correlations between all measures. Notably, all variables in the study were highly and—with the exception of number line estimation—positively correlated. High correlations between variables are consistently found within the literature (Hawes et al. 2019) and serve as further justification for investigating whether these foundational skills are uniquely predictive of mathematics achievement.

### 3.2. Structural Equation Models

#### 3.2.1. Measurement Models

As noted earlier, we initially constructed a four-factor measurement model, which included latent constructs for all three cognitive foundation skills as well as a fourth latent construct of mathematics achievement that consisted of arithmetic and number line estimation. However, model fit indices indicated poor model fit. As a result, we tested a three-factor model including constructs for numerical, EF, and spatial skills and analyzed number line estimation and arithmetic achievement as separate outcome measures. 

#### 3.2.2. Structural Models 

Given the high correlations between variables, there was concern that a general intelligence factor (*g*-factor) might better explain the data than the three-factor models tested above. To rule out this possibility, we modelled the data using a single general intelligence variable to predict children’s number line estimation and arithmetic performance. We then compared these models to our three-factor models reported above. In each case, the three-factor model provided a better fit than a single *g*-factor model. That is, for number line estimation, the three-factor model was a better fit (*AIC* = 7643.43, *BIC* = 7721.08) than the intelligence model (*AIC* = 7699.96, *BIC* = 7762.07). For arithmetic, the three-factor model was a better fit (*AIC* = 8868.68, *BIC* = 8946.63) than the intelligence model (*AIC* = 8921.45, *BIC* = 8983.81). Overall, these findings suggest that while numerical, EF, and spatial skills are all highly related, they represent unique constructs and provide a better explanatory model of children’s mathematics performance (number line estimation and arithmetic) than a model of general intelligence.

Separate structural models were created to examine how latent factors representative of numerical, EF, and spatial skills related to (1) number line estimation, (2) arithmetic performance, (3) arithmetic strategy use as mediator of cognitive skills and arithmetic accuracy, and (4) the likelihood of using each arithmetic strategy. As shown in Table 3, the model fit indices for each one of these analyses suggest good model fit on several key metrics. The specific findings related to each model are reported next. Importantly, model fits were poor (below the suggested criterion) when we included age; therefore, all models exclude age. We address this limitation further in the discussion. 

#### 3.2.3. Number Line Estimation 

**Figure 2:**
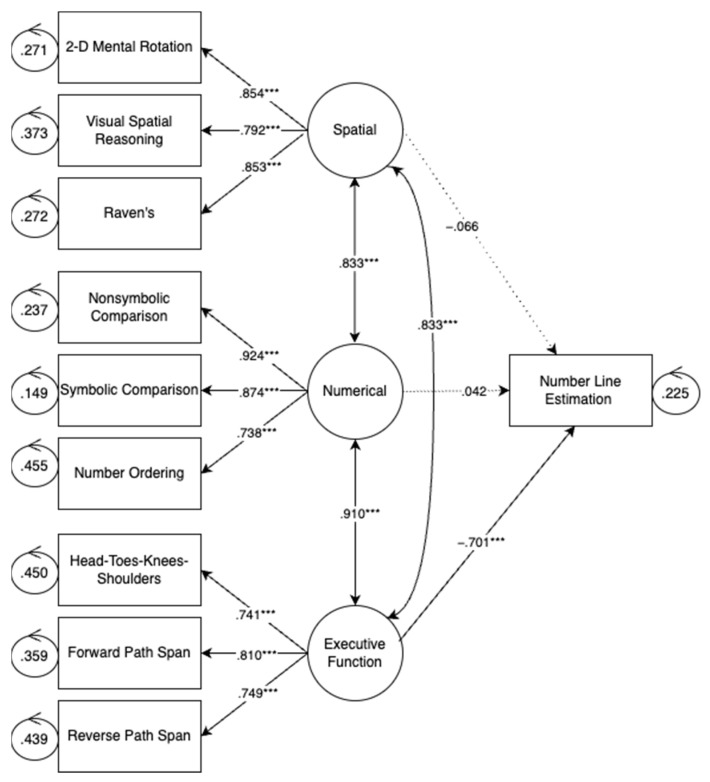
Number line estimation model. Note: *p* < 0.001 ***.

Figure 2 shows the relations between each latent variable—numerical, EF, and spatial—and number line estimation. As shown in Figure 2, only executive function skills were significantly related to number line estimation, controlling for children’s numerical and spatial skills. This finding is contrary to previous work which has found that spatial skills are predictive of number line estimation (Gunderson et al. 2012; LeFevre et al. 2013). 

#### 3.2.4. Arithmetic Accuracy 

**Figure 3:**
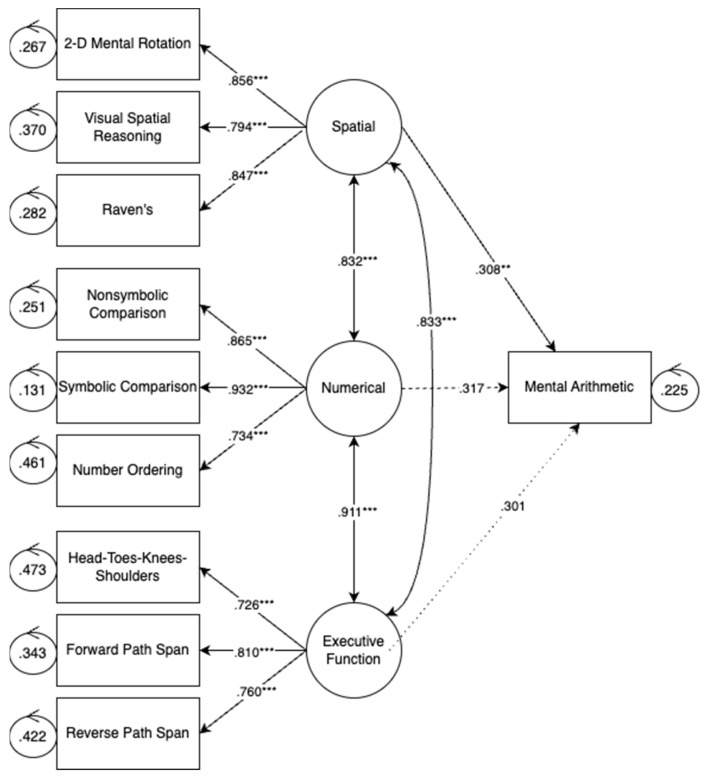
Arithmetic performance model. Note: *p* < 0.01 **, and *p* < 0.001 ***.

Figure 3 shows the relations between each latent variable—numerical, EF, and spatial—and arithmetic accuracy. As shown in Figure 3, only spatial skills were significantly related to arithmetic accuracy, while controlling for children’s numerical and EF skills. Numerical skills were only marginally related to arithmetic accuracy (*p* = 0.055), when controlling for spatial and EF skills. These findings align with Hawes et al. (2019), who found that spatial skills were the strongest predictor of both numeration and geometry. 

#### 3.2.5. Mediation Model—Arithmetic Strategy Use

**Figure 4:**
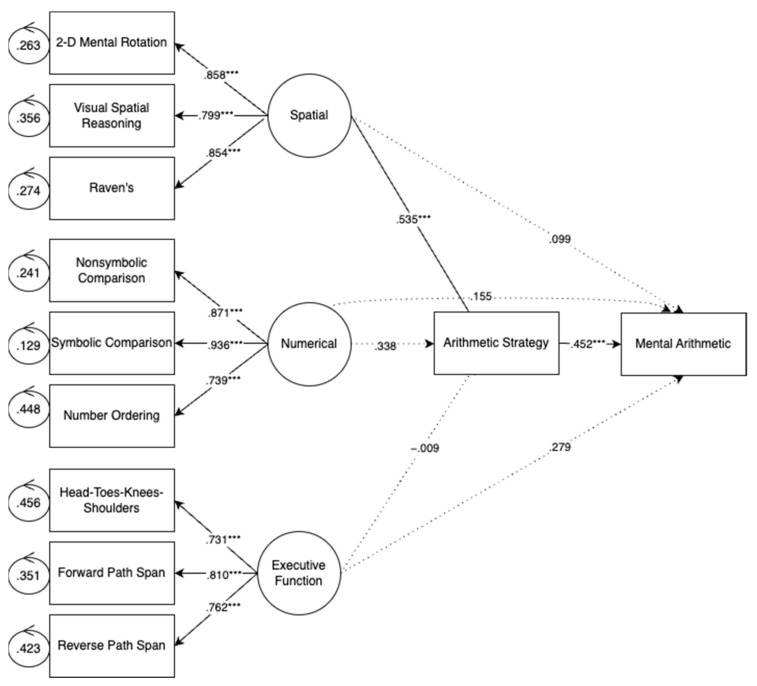
Arithmetic strategy as a mediator of the relations between cognitive factors and arithmetic accuracy. Note: *p* < 0.001 ***.

Figure 4 shows the results of a mediation model where children’s strategy is tested as a mediator of the relations between each cognitive factor and arithmetic accuracy. This model reveals that strategy is predicted only by spatial skills and strategy is the only predictor of arithmetic performance. In other words, strategy fully mediates the relationship between spatial skills and arithmetic performance. This is in line with existing research which suggests that spatial skills are predictive of strategy use (Casey et al. 2017; Laski et al. 2013). 

#### 3.2.6. Relations between Cognitive Factors and Strategy Use

In order to further probe how arithmetic strategy was related to foundational skills and arithmetic performance, we constructed a post-hoc structural equation model where we looked at how latent factors for foundational skills were predictive of different strategy types. Here, strategy types are operationalized as the percentage of times a child used the given strategy (e.g., a child who used decomposition 33% of the time would have a decomposition score of 33). None of the foundational skills were predictive of guessing, count up, or count on (lower addend) strategies. Numerical skills were marginally predictive of count on (higher addend; *β* = 0.537, *p* = 0.050). Spatial skills were predictive of decomposition (*β* = 0.438, *p* = 0.016) and retrieval (*β* = 0.619, *p* < 0.001) strategies. These results are in line with our overall strategy model as well as previous findings that spatial skills are predictive of decomposition and retrieval specifically (Casey et al. 2017; Laski et al. 2013). 

## 4. Discussion

This study examined how numerical, EF, and spatial skills related to two mathematics achievement measures: number line estimation and arithmetic performance. In line with previous work by Hawes et al. (2019), we found that all three skills were highly related, yet separable, latent constructs. Moreover, the three-factor model (i.e., modelling numerical, EF, and spatial skills as separate latent variables) fit the data better than a general intelligence (*g*-factor) model. While EF skills were the only unique predictor of number line performance, spatial skills were the only unique predictor of arithmetic (addition) performance. Additionally, spatial skills were related to the use of more advanced addition strategies (e.g., composition/decomposition and retrieval), which in turn were related to children’s overall arithmetic performance. In other words, children’s strategy use fully mediated the relation between spatial skills and arithmetic performance. Whereas numerical skills were marginally associated with children’s likelihood of using the *min* strategy (counting on from the larger addend; e.g., for the problem 3 + 5, a child would say, “5 … 6, 7, 8”), spatial skills were uniquely related to the use of composition/decomposition strategies (e.g., for 5 + 4, a child might say “I know 4 + 4 is 8, so 1 more is 9”) and automatic retrieval (e.g., just knowing that 4 + 4 = 8). 

### 4.1. Number Line Estimation 

Children’s number line performance was uniquely predicted by EF skills, over and above numerical and spatial skills. This is a surprising finding, as prior research has suggested that both numerical and spatial skills play a fundamental role in number line estimation (Gunderson et al. 2012; LeFevre et al. 2013). In comparison, the role of EF skills has received less attention. One explanation for the current finding is that EF skills, such as inhibitory control and self-monitoring, may play an important role in assessing the accuracy of one’s initial estimate. Said differently, while numerical and spatial skills may help children make a quick and approximate magnitude estimate, EF skills may further assist in making fine-tuned adjustments to one’s initial estimate. For example, when asked to locate 67 on a 0–100 number line, a child may initially reason that “67 is somewhere between 50 and 100”, but, upon further reflection, more precisely reason that “67 is located just less than 70”, all the while, using proportional/spatial reasoning to locate the corresponding numerical positions on the line/scale. In this way, higher EF skills may be related to a higher propensity to integrate numerical and spatial strategies, override or fine-tune initial estimates and, ultimately, lead to more accurate performance. Moving forward, process-based accounts of number line performance are needed, taking into account the precise ways in which children’s numerical, EF, and spatial skills influence strategy choice and the specific decision-making processes involved with number line estimation (e.g., see Dotan and Dehaene 2013; Dotan et al. 2019). 

### 4.2. Arithmetic Performance and Strategy Use 

After taking into account children’s numerical and EF skills, only spatial skills explained unique variance in arithmetic (addition) accuracy. A primary purpose of this study was to test the extent to which the findings of Hawes et al. (2019) extended to different measures of mathematics. While Hawes et al. (2019) found that spatial and, to a lesser extent, numerical skills, were unique predictors of mathematics, there was concern that these findings may have been due to the way in which mathematics was assessed; that is, through geometry and numeration measures that featured novel/unfamiliar problems and presented through the use of visual–spatial representations (e.g., reasoning about graphs, shapes, arrays, etc.), with many items requiring explicit spatial reasoning (e.g., identifying a figure given the front, side, and top views). The present finding suggests that spatial skills also play an important role in mathematics tasks that are more familiar to children and that are not overtly spatial (at least on the surface).

This raises the question of how spatial skills might support children’s arithmetical reasoning. To gain insights into this question, we measured children’s strategy use. Perhaps unsurprisingly, given the findings just reported, we found that strategy use mediated the relations between spatial skills and overall addition accuracy. That is, higher spatial skills were related to the use of more sophisticated addition strategies, which in turn, was related to higher overall accuracy. A follow-up analysis revealed that spatial skills were unrelated to lower-level addition strategies (e.g., counting all) and uniquely related to higher-level strategies of composition/decomposition and retrieval strategies. This finding is consistent with previous research showing that children’s spatial skills share both concurrent and longitudinal relations with the use and development of composition/decomposition and retrieval strategies (Casey et al. 2017; Laski et al. 2013). 

As previously theorized, it is possible that the mental operations that underlie the composition/decomposition of shapes (as measured in the current study) may also serve the composition/decomposition of number (see Casey and Fell 2018; Mix 2019). Moreover, more advanced addition strategy use may be related to spatial visualization skills and the important role they play in one’s ability to recall, mentally organize, visualize, and manipulate the problem at hand. These possibilities most closely align with the *spatial modelling account* (Hawes and Ansari 2020). Critically, our findings also provide counter evidence to the *working memory account*, which postulates that space-mathematics relations may best explained by children’s working memory, EF, or general intelligence skills. In the present study, neither EF skills, including measures of working memory, or a *g*-factor model, were able to explain the space-mathematics relation as well as the relations between children’s spatial visualization skills and addition accuracy and strategy use. Moving forward, more targeted efforts are needed to further reveal the mechanisms linking spatial visualization skills and arithmetic performance. 

Although children’s numerical skills were not related to addition accuracy, they were related to the use of the counting on from the larger addend strategy (albeit with a *p* value of 0.05). This finding supports the importance of being able to quickly and accurately access the numerical magnitudes/cardinality of each addend (e.g., 5 + 3) in order to engage in the counting-on strategy. So, while this finding is perhaps unsurprising, it does offer further evidence that children’s numerical, EF, and spatial skills provide both shared and unique pathways to mathematics, and, moreover, that these relations vary depending on the specific mathematics at hand. 

### 4.3. Limitations and Future Directions 

One major limitation of the current study was that we were unable to account for age and grade in our current models. We constructed models accounting for age and grade (separately) at the level of cognitive foundations and at the level of mathematics achievement, and we created multi-group models to account for age; however, many of these models did not converge and those that did had very poor fits (i.e., outside the suggested criterion). As such, we did not account for these important developmental variables within our models. We have some confidence in our models given that previous work investigating EF, numerical, and spatial skill using a largely overlapping sample (with greater statistical power) found that age did not fully account for the relations observed (Hawes et al. 2019). That said, future work that addresses differences in foundational skills and mathematics achievement across age or grade is certainly required in order to fully understand the relations among these domains.

Notably, this work is also cross-sectional in nature, and therefore we cannot draw any conclusions regarding the directionality of mediation analyses. Investigating how cognitive foundational skills such as numerical, EF, and spatial skills longitudinally relate to mathematics achievement is an important future direction (see Verdine et al. 2017 for some progress in this regard). Additionally, it is critical to consider that all variables measured in the study were very highly correlated. Therefore, while we are confident that these cognitive foundational skills are robustly predictive of mathematics achievement, further replication remains necessary in order confirm the specific relations between foundational skills and specific measures of mathematics achievement. 

Finally, we wish to point out that the current study used eleven instruments to assess numerical, spatial, EF, and mathematical skills. We consider this as both a strength and limitation of our work. On one hand, the creation of latent factors is statistically sound and the use of multiple measures to construct those latent factors is required. On the other hand, this approach is not feasible for use by practitioners. It is essential that future work investigates and considers which of these, if any, are the most robust predictors of the various skills and how they could be used to inform mathematics teaching and intervention efforts.

## 5. Conclusions

Our results indicate close relations between children’s numerical, EF, spatial, and mathematics skills. These results are consistent with a cognitive foundations model, in which the relations between each cognitive skill and mathematics performance are likely to depend on the mathematics task or learning process under consideration. We found especially strong relations between EF skills and number line estimation and between spatial skills and arithmetic performance and strategy use. These findings are meaningful insofar as both number line estimation and arithmetic have been identified as robust predictors of later mathematics achievement (Schneider et al. 2018; Lin and Powell 2022). Moving forward, it will be important to continue strengthening our understanding of the dynamic interplay of these foundational cognitive skills and their relations with various branches of mathematics. Such efforts have the potential to inform mathematics assessment and instruction by providing key insights into the cognitive bases of individual differences in children’s mathematics thinking and learning.

## Figures and Tables

**Table 1:** Description of measures used in the study.

Measures	Task Description	Example Items
**Numerical Measures**		
Symbolic Number Comparison	Participants select the numerically larger of two Hindu-Arabic numerals 1 min to complete as many items as possible	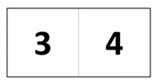
Nonsymbolic Number Comparison	Participants select the numerically larger of two dot arrays 1 minute to complete as many items as possible	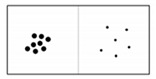
Ordering	Participants indicate whether or not a sequence of numerals are in numerical order 1 min to complete as many items as possible	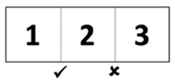
**Executive Function Measures**		
Head-Toes-Knees-Shoulders	Participants touch the opposite body part of the one instructed	“*When I say touch your* *head, I really want you* *to touch your toes*”
VSWM - Forward Path Span	Participants are presented with a random sequence of green dots on an iPad screen and watch as individual dots light up one at a time Participants recall the exact sequence	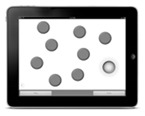
VSWM - Reverse Path Span	Participants are presented with a random sequence of green dots on an iPad screen and watch as individual dots light up one at a time Participants recall the exact sequence but in reverse order in which they occurred	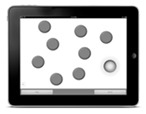
**Spatial Measures**		
Visual-Spatial Reasoning	Participants are presented with 4 different types of ‘spatial puzzles’ requiring participants to visualize solutions to partially completed puzzles, composition/decomposition tasks, and mental paper folding challenges	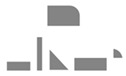 “*Which three pieces will go* *together to make the shape* *above?”*
2D Mental Rotation	Participants select amongst four options a given shape that can be made by mentally rotating and translating two separated shapes	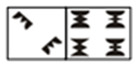
Raven’s Matrices	Participants are presented with a partially completed image or visual-spatial pattern and must select amongst 6 options the piece that best completes the image/pattern	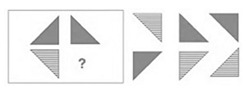
**Mathematics Measures**		
Addition	12 addition problems of increasing difficulty presented to child aurally	e.g., 2 + 1 … 8 + 7
Number Line	Participants are given an empty number line bounded by 0 and 10 or 100 (depending on the age of the children) and asked to indicate the locations of different numbers (e.g., where does 9 go?)	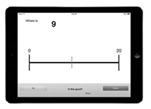

Note that for copyright reasons, the example item for Raven’s matrices was reproduced and does not constitute a direct replica of an actual test item. VSWM = visual-spatial working memory.

**Table 2:** Descriptive statistics for all measures.

	N	Mean	SD	Min	Max	Skew	Kurtosis
Visual Spatial Reasoning (out of 20)	180	9.23	3.62	2.00	19.00	0.59	−0.11
2-D Mental Rotation (out of 16)	180	8.08	3.38	1.00	16.00	0.12	−0.76
Raven’s Matrices (out of 36)	180	18.13	6.20	4.00	33.00	−0.04	−0.45
Nonsymbolic Comparison (timed task)	180	12.98	7.49	−4.00	34.00	−0.16	−0.67
Symbolic Comparison (timed task)	180	17.73	11.91	−5.00	47.00	0.07	−0.85
Number Ordering (timed task)	178	6.33	5.54	−7.00	20.00	−0.02	−0.48
Head-Toes-Knees-Shoulders (out of 40)	179	27.24	11.19	0.00	40.00	−1.22	0.26
Forward Path Span	172	3.66	2.27	0.00	11.00	0.27	−0.48
Reverse Path Span	173	2.61	2.18	0.00	9.00	0.77	−0.30
Number Line Estimation (out of 1)	172	0.17	0.11	0.03	0.55	1.33	1.74
Mental Arithmetic Score (out of 12)	175	6.76	4.46	0.00	12.00	−0.39	−1.43
Mental Arithmetic Strategy (out of 6)	166	3.08	1.83	0.00	6.00	−0.23	−1.20
Age	180	6.21	1.38	4.08	9.17	0.19	−1.05
Grade	180	3.13	1.78	1.00	6.00	0.08	−1.50

**Table 3:** Model fit statistics for each structural model.

Metric of Model Fit	Measurement Model *	Number Line Estimation Model	Arithmetic Accuracy Model	Arithmetic Strategy Mediation Model	Likelihood Arithmetic Strategy Model	Criterion
Robust Chi-Squared	0.010 (*df* = 24)	0.000 (*df* = 45)	0.032 (*df* = 30)	0.025 (*df* = 36)	0.000	*p* > 0.05
Chi-Squared with Satorra-Bentler scaling correction factor	--	1.052	1.055	0.999	0.885	
Robust Root Mean Square of Error Approximation (RMSEA)	0.069	0.076	0.058	0.057	0.053	<0.10
Robust Standardized Root Mean Square	0.030	0.033	0.029	0.029	0.034	<0.08
Robust Comparative Fit Index (CFI)	0.981	0.974	0.986	0.987	0.979	>=0.95

Note. Criterion values follow the guidelines set out by Kline (2015). * Measurement model statistics are not robust.

## Data Availability

Data are available upon request.
